# Baroreflex mechanisms and response to exercise in patients with heart disease

**DOI:** 10.1111/j.1475-097X.2012.01127.x

**Published:** 2012-03-26

**Authors:** Nagaharu Fukuma, Kazuyo Kato, Kazuo Munakata, Hiroko Hayashi, Yuko Kato, Noriko Aisu, Hiroshi Takahashi, Kousuke Mabuchi, Kyoichi Mizuno

**Affiliations:** Cardiology Department of internal medicine, Nippon Medical SchoolTokyo, Japan

**Keywords:** baroreflex, exercise, exercise tolerance, heart rate, sympathetic nerve

## Abstract

**Background:**

Past reports showed that the baroreflex continuously regulates hemodynamics during exercise. However, it is still clinically unclear. If baroreflex mechanism is able to influence actually exercise cardiovascular control, baroreflex sympathetic and/or parasympathetic function relates to response to exercise. Therefore, we examined the relationship of heat rate changes to both blood pressure increment and decrement with tolerance and chronotropic response to peak exercise in patients with heart disease.

**Methods:**

In 25 male heart disease patients (60 ± 9 years) without decompensated heart failure, baroreceptor reflex sensitivity (BRS ms mmHg^−1^) was measured by reflex heart rate responses to changes in blood pressure after phenylephrine (P-BRS) and nitroglycerin (N-BRS) injection, respectively. Symptom-limited treadmill exercise test was performed according to Bruce's protocol.

**Results:**

(i) The absolute values of blood pressure change after the administrations were similar between the agents because the dosages of nitroglycerin and phenylephrine were set to equalize absolute changes in blood pressure. (ii) In this study population, the ratio of N-BRS to P-BRS was not significantly correlated with hypertension and diabetes mellitus. (iii) Exercise capacity (METs) (*r* = −0·626) and heart rate response to exercise per METs (*r* = 0·670) was significantly related to N-BRS but not to P-BRS.

**Conclusion:**

We found that the abnormality of baroreflex function in the presence of blood pressure decrements can lead to insufficient capacity and easy sympathetic activation during exercise.

## Introduction

Many studies of the baroreceptor reflex have revealed various physiological effects and elucidated that the baroreflex regulates hemodynamics not only at rest but also during exercise (Melcher & David, 1981). According to animal experiments (Walgenbach & Donald, 1983; Rowell & O'Leary, 1990a), the roles of baroreflex during exercise involve the activation of sympathetic system to a level necessary to adapt to exercise stress through the resetting of operating point. Therefore, the baroreceptor reflex is thought to be an important mechanism for sympathetic activation during exercise. However, this has not been clarified by a clinical investigation.

In this study, based on clinical examination, we evaluated the sympathetic and parasympathetic functions through baroreceptor reflex system at rest and examined whether these mechanisms relate to response to exercise. The motive is that we hypothesized that baroreflex function at rest has a relationship with that during exercise and that abnormal baroreflex sympathetic acceleration during exercise leads to exercise intolerance and disturbed chronotropic response. Furthermore, these phenomena are reported to be associated with a poor prognosis in patients with heart disease (Lauer *et al*., 1996). Thus, an investigation into the alteration of sympathetic response to exercise in patients with heart disease was thought to be clinically important. Actually, we estimated the heart rate response to blood pressure decrements after administration of a vasodilator according to a previously reported procedure (Olivari *et al*., 1983) to assess the parameter reflected mainly by sympathetic excitability through the baroreflex mechanism (Sundlöf & Wallin, 1978; Sanders *et al*., 1989). Then, the relationship between the responsiveness using a vasodilator and adaptation ability including chronotropic response to exercise was studied in comparison with the conventional method using phenylephrine (Smyth *et al*., 1969), and the clinical differences between the two kinds of baroreflex index using the ratio of baroreflex sensitivity after nitroglycerin to that after phenylephrine were clarified.

## Methods

### Study population

Study population consisted of 25 men [mean age 60 ± 9 years (mean ± SD)] with stable ischaemic heart disease without exercise-induced ischaemia and decompensated heart failure. Ten subjects had been treated for myocardial infarction and 15 for angina pectoris. Excluded were those who had a myocardial infarction within 3 months prior to the study, who were more than 70 years old or who had resting blood pressure >160/90 mmHg. Subjects were eligible for study if exercise was only limited by symptoms of fatigue or dyspnoea but not by angina, syncope or claudication. Other exclusion criteria included the use of β-blockers or non-dihydropyridine calcium channel blockers such as diltiazem or verapamil. All aspects of the study were carefully explained to study subjects before informed consent for participation was given.

### Exercise treadmill testing

All subjects performed symptom-limited treadmill exercise testing according to the standard Bruce protocol (Sheffield, 1975). Exercise was stopped by symptoms of fatigue or dyspnoea when patients reached a rating of 17 on the Borg perceived exertion scale (Borg, 1982). Heart rate (beats per min) and 12-lead electrocardiogram were monitored continuously during exercise using the Case 15 Stress System (Marquette Electronics, Inc., Milwaukee, WI, USA). Blood pressure (mmHg) was measured every minute with an automatic sphygmomanometer (STBD-780B; Nihon Colin Co., Ltd., Aichi, Japan). A metabolic equivalent at peak exercise (METs ml kg^−1^ min^−1^) was estimated using the formula described by the American College of Sports Medicine (Ehrman, 2010). This formula is expressed as:


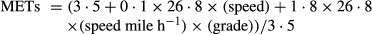


### Baroreflex sensitivity (BRS)

Baroreceptor reflex sensitivity (BRS) was assessed by bolus phenylephrine (Sanders *et al*., 1989) and nitroglycerin injection through cubital vein, respectively. The subjects were placed in a supine position in a comfortable environment for at least 30 min. Electrocardiograms were monitored by continuous recording of lead II. Blood pressure was measured from the radial artery on the side opposite the intravenous line using tonometry apparatus for the non-invasive continuous blood pressure monitoring, Jentow, (Nihon Colin Co., Ltd.) during the entire test. After the stabilization period, patients received bolus administration of phenylephrine (2·0–4·0 μg kg^−1^) or nitroglycerin (0·5–1·0 μg kg^−1^) to change the systolic blood pressure by 15–20 mmHg. The bolus injection was repeated at least three times at whatever dose was found to be efficacious. Then, the linear regressions of the R-R intervals and systolic blood pressure were calculated, including all of the points between the beginning of the first significant increase in systolic arterial pressure and the end of the plateau of systolic arterial pressure. If the correlation coefficients were statistically significant (*r* = 0·6, *P*<0·05), test results were used for the analysis. The final slope of these regression lines was the mean value of at least three tests and was considered to be an index of BRS, which was expressed in units of milliseconds per mmHg (ms mmHg^−1^).

In all subjects, two types of BRS were measured separately. We designated BRS using phenylephrine as P-BRS and BRS using nitroglycerin as N-BRS. We classified the study population into two groups: the low N-BRS/P-BRS ratio group, which included 12 subjects with a ratio <0·5, and the high N-BRS/P-BRS group, which included 13 subjects with a ratio of 0·5 or more. Then, we investigated the differences in clinical background between the two groups.

### Statistical analysis

All values are expressed as mean ± SD. We investigated the relationship between BRS and the index of response to peak exercise using Pearson's correlation coefficient. Comparisons of parameters between groups were performed using the unpaired Student's *t*-test and chi-square analysis. Differences were considered statistically significant at *P*-values <0·05.

## Results

Table 1 shows the responses of systolic blood pressure and the R-R interval on electrocardiogram to phenylephrine and nitroglycerin administration, respectively. Absolute change in blood pressure during the P-BRS test did not differ from that during the N-BRS test. However, the change in the R-R interval was smaller with the N-BRS test than with the P-BRS test.

**Table 1:** Respective response of systolic blood pressure and heart rate to phenylephrine and nitroglycerin administration

	After phenylephrine	Nitroglycerin	*P*-value
Change in SBP, mmHg	18 ± 7	16 ± 4	ns
Change in R-R interval, ms	132 ± 42	81 ± 59	<0·05
BRS, ms mmHg^−1^	5·5 ± 3·8	3·2 ± 1·7	<0·01

BRS, baroreflex sensitivity; ns, not statistically significant.

Data are expressed as mean ± SD. Change in SBP, Absolute value of maximum change in systolic blood pressure during vasoactive agent administration. Change in R-R interval, Absolute value of maximum change in the interval of *R* wave on electrocardiogram after the administration. *P* value was calculated between the responses to phenylephrine and nitroglycerin.

Table 2 shows the comparisons of clinical characteristics and exercise test results between low and high ratios of N-BRS to P-BRS. The presence of diabetes mellitus or hypertension did not differ between the two groups. However, the higher ratio group had a tendency towards greater age and a low left ventricular ejection fraction. Exercise responses of HR and systolic blood pressure had no significant differences between the two groups.

**Table 2:** Clinical characteristics and N-BRS to P-BRS ratio

N-BRS/P-BRS ratio	<0·5, *n* = 12	≥0·5, *n* = 13	*P*-value
Age, year old	56 ± 9	63 ± 9	<0·1
Complication, (%)			
Diabetes mellitus	2 (17%)	6 (46%)	ns
Hypertension	4 (33%)	6 (46%)	ns
Low EF	1 (8%)	5 (38%)	<0·1
Treadmill exercise test			
Change in SBP, mmHg	52 ± 22	52 ± 25	ns
Heart rate, beats per min	55 ± 13	57 ± 12	ns

Comparison of clinical characteristics between two groups divided by 0·5 of N-BRS to P-BRS ratio; N-BRS, baroreceptor reflex sensitivity calculated by the heart rate response to blood pressure decrement after nitroglycerin administration; P-BRS, baroreceptor reflex sensitivity calculated by the response to blood pressure increment after phenylephrine administration; Low EF, Low left ventricular ejection fraction defined by <40%.

**Figure 1:**
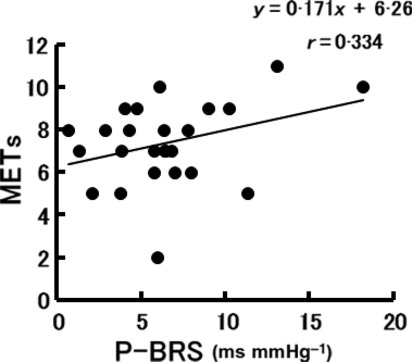
Relationship between P-BRS and exercise tolerance. This linear regression line showed a weak positive coefficient of correlation, but not significant (*P*<0·1). P-BRS, baroreceptor reflex sensitivity evaluated by phenylephrine; METs, metabolic equivalents.

**Figure 2:**
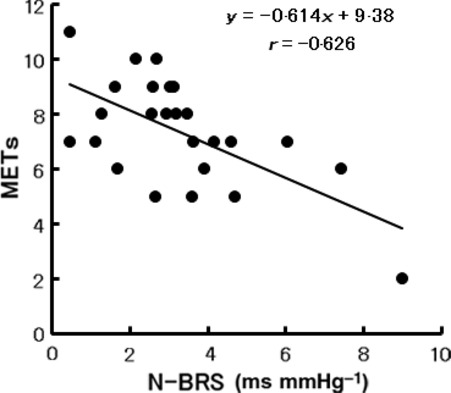
Relationship between N-BRS and exercise tolerance. This linear regression line showed an inverse and closer correlation (*P*<0·01) compared with the line of P-BRS. N-BRS, baroreceptor reflex sensitivity evaluated by nitroglycerin; METs, metabolic equivalents.

**Figure 3:**
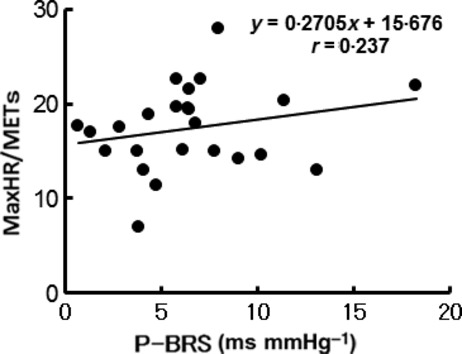
Relationship between P-BRS and heart rate response through exercise stress. Linear regression line did not reveal significance. P-BRS, baroreceptor reflex sensitivity evaluated by phenylephrine; maxHR/METs, heart rate at peak exercise to metabolic equivalents ratio.

**Figure 4:**
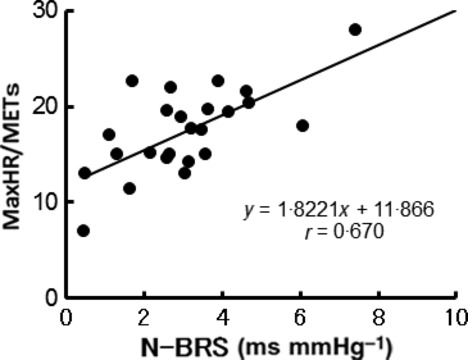
Relationship between N-BRS and heart rate response per exercise stress. This linear regression line indicated closer positive regression line (*P*<0·01) in comparison with that of P-BRS. N-BRS, baroreceptor reflex sensitivity evaluated by nitroglycerin; maxHR/METs, heart rate at peak exercise to metabolic equivalents ratio.

P-BRS had only a weak tendency of a positive correlation with exercise tolerance (METs), as shown in Fig. 1. However, N-BRS was related inversely and closely with METs, as shown in Fig. 2. We investigated whether the baroreceptor reflex function relates the heart rate response to peak exercise. The heart rate response was assessed by the ratio of maximum heart rate to maximum load (maxHR/METs) as an index of responsiveness to exercise stress. P-BRS had a weak tendency for a positive correlation with maxHR/METs (Fig. 3). However, N-BRS had a significant positive coefficient of correlation with maxHR/METs (Fig. 4).

## Discussion

In the past, it was noted that the baroreflex function was suppressed during exercise to permit sympathetic excitation. However, later animal studies (Melcher & David, 1981) showed that the baroreflex has a forceful role in sympathetic excitation. The stimulation of motor muscles during exercise activates mechano- and metabo-reflex systems, and baroreflex regulation should shift the set point of the target blood pressure to the higher side (Plotts *et al*., 1993; Plotts & Mitchel, 1998; Miki *et al*., 2003; Smith *et al*., 2003). This shifting leads to the necessary level of sympathetic tone (Rowell & O'Leary, 1990b).

In this study, we examined the baroreflex heart rate response to change in blood pressure not only after phenylephrine administration but also after nitroglycerin administration. BRS is most generally measured using phenylephrine (Sanders *et al*., 1989). However, several studies of BRS using nitrate have been described (Ferguson *et al*., 1992). Although N-BRS is developed through both sympathetic and parasympathetic pathways, N-BRS is reported to be mainly constructed by sympathetic contribution (Sundlöf & Wallin, 1978; Sanders *et al*., 1989). In research on differences between BRS using phenylephrine and nitrate, the ratio of BRS with nitroprusside to that with phenylephrine was examined and it was revealed that the ratio reflected the pathophysiological condition of heart failure (Olivari *et al*., 1983). This difference is speculated to be related to the potential ability of the autonomic system to adapt to stress. For example, in cases of severe heart failure, the sympathetic tone is activated sufficiently at rest before the baroreflex test with nitrate and results in the low BRS. Thus, the baroreflex test using vasoactive agent is thought to be able to reflect not only the sensitivity of the baroreflex system, but also the reserve capacity of the autonomic system to adapt to stress.

Our current results showed that the both exercise tolerance (METs) and heart rate response per METs are closely correlated with the ability of tachycardic response to blood pressure decrements after nitrate in comparison with that of phenylephrine, although the absolute change in blood pressure was similar in both baroreflex tests. These phenomena suggest that the easy excitability of sympathetic tone in N-BRS test as a pathophysiological reflection of heart disease limits exercise performance and induces intense chronotropic response in low exercise load. And it is proposed that there was the similarity of baroreflex mechanism between N-BRS test and intense exercise. If the baroreflex function with nitroglycerin is accelerated, it is thought that optimal sympathetic excitation cannot be acquired during exercise. This speculation has potential clinical implications. As revealed in this study, the inappropriate sympathetic activation to stress leads to exercise intolerance and to rapid tachycardic response from early exercise stage. This abnormal adaptation for exercise is an independent predictor of prognosis in heart disease (Bruce *et al*., 1974; Chin *et al*., 1979). It is possible to show that BRS with nitrate can predict mortality and morbidity in heart disease through a different way from phenylephrine method (La Rovere *et al*., 1998).

A weak correlation between P-BRS and chronotropic response to exercise was also indicated, although the interaction of blood pressure with heart rate is dissimilar between the exercise and baroreflex test using phenylephrine. The reflection of P-BRS during exercise could be interpreted as mainly mediated by the parasympathetic mechanism. In past reports, it was shown that BRS with phenylephrine indicates a parasympathetic tone (Farrell *et al*., 1991) and that the rapid attenuation of parasympathetic tone induces a tachycardic response in the early phase of exercise (Arai *et al*., 1989). This pathway from P-BRS to parasympathetic withdrawal is thought to be the reason. Furthermore, as a mechanism other than parasympathetic function, there is the indirect effect of the baroreflex system on chronotropic response. We previously showed that BRS with phenylephrine is related to the heart rate response at a later phase of exercise accompanied by sympathetic acceleration (Fukuma *et al*., 2004). In our article, we speculated that the inverse interaction between baroreflex and chemoreflex function leads to this phenomenon via an indirect sympathetic mechanism (Ponikowski *et al*., 1992; Ichinose *et al*., 2002). However, BRS with phenylephrine cannot be sufficiently explained by sympathetic acceleration at peak exercise. From the standpoint of the baroreflex function, present investigation is the first report to examine more closely the relationship between baroreflex sympathetic excitability and exercise performance.

This study has several limitations. One concerns the study population. For example, we recruited only patients with stable heart disease, but not with severe conditions such as decompensated heart failure. We need to additionally examine baroreflex function in patients with various states of heart disease and in healthy subjects. Another limitation concerns the measurement of baroreflex function. We assessed BRS during bed rest, although baroreflex gain can be altered during exercise. During exercise, however, it is difficult to measure BRS with Jentow technology. Furthermore, we cannot choose direct recording of arterial blood pressure because of invasiveness. The baroreflex system was shown to continue to function after the start of exercise, while the set point of the target blood pressure is shifting (Melcher & David, 1981). Therefore, we believe that BRS, which is measured at rest, also reflects the baroreflex function during exercise. If new non-invasive and accurate methods are developed, BRS should be tested during exercise in clinical studies of human subjects.

Here, we examined sympathetic pathways from the baroreflex mechanism to the adaptation response to exercise although indirectly. We found that the acceleration of baroreflex function in the presence of blood pressure decrements can lead to insufficient exercise tolerance and easy sympathetic activation during low-intensity exercise, and it is possible to predict the chronotropic incompetence and poor prognosis in patients with heart disease.
